# Broad complex tachycardia; never judge a book by its cover

**DOI:** 10.1007/s12471-020-01496-w

**Published:** 2020-10-02

**Authors:** M. V. Regeer, L. F. Tops, M. de Riva Silva

**Affiliations:** Department of Cardiology, Leiden Heart-Lung Center, Leiden University Medical Center, Leiden, The Netherlands

## Answer

**Fig. 1:**
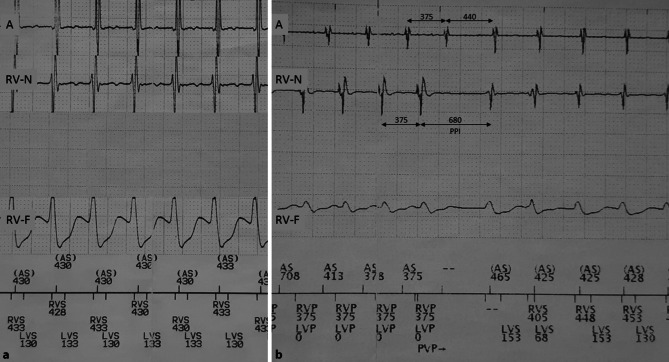
**a** Implantable cardioverter defibrillator tracing. **b** Unsuccessful overdrive pacing with relatively long post-pacing interval (*A* atrial lead electrogram, *RV‑N* right ventricular lead near-field electrogram, *RV‑F* right ventricular far-field electrogram, *PPI* post-pacing interval)

The electrocardiogram (ECG) at admission showed a broad complex tachycardia of 138 bpm with superior axis, left bundle branch block (LBBB) morphology and a transition in lead V5. There was no apparent atrioventricular (AV) dissociation. His device for cardiac resynchronisation therapy with defibrillator function (CRT-D) was interrogated and showed a regular tachycardia with 1:1 AV relationship and a short ventriculoatrial (VA) time (<60 ms, not compatible with a concealed bypass) (Fig. 1a).

Ventricular overdrive pacing was performed from the right ventricular (RV) lead (located at the RV apex) at 94%, 88% and 84% of the tachycardia cycle length. With the RV bursts at 94% and 88% of the tachycardia cycle length, the tachycardia did not terminate but was entrained. Fig. 1b shows the response to entrainment with a ventricular-atrial-ventricular (V-A-V) response and a long post-pacing interval. At 84% of the cycle length, the arrhythmia was terminated (unlikely for atrial tachyarrhythmia).

**Fig. 2:**
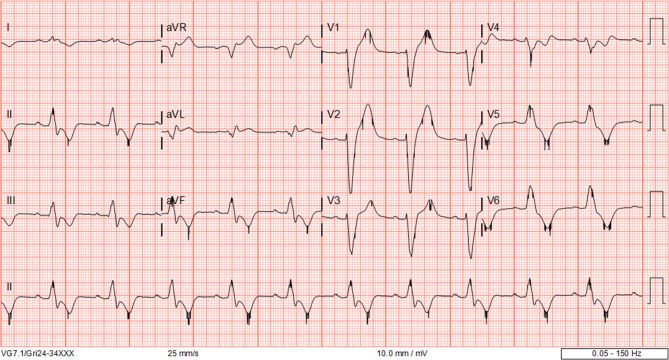
Electrocardiogram without pacing

An electrocardiogram without biventricular pacing (Fig. 2) showed sinus rhythm with a broad LBBB with identical QRS morphology as the tachycardia. In conclusion, there was a broad complex tachycardia without AV dissociation, a QRS morphology identical to non-paced conducted sinus rhythm, a short VA interval and a long post-pacing interval as a result of entrainment from the RV apex, all findings compatible with AV nodal re-entry tachycardia.

The metoprolol dosage was increased and the patient was instructed to perform Valsalva manoeuvre in case of palpitations. Alternative treatment with a calcium channel blocker was less opportune regarding the negative inotropic effects, preferably avoided in this patient with non-ischaemic cardiomyopathy. In case of recurrence, ablation will be considered.

